# The transcription factor USF1 promotes glioma cell invasion and migration by activating lncRNA HAS2-AS1

**DOI:** 10.1042/BSR20200487

**Published:** 2020-08-21

**Authors:** Juntong Wang, Jingshun Gu, Aiwu You, Jun Li, Yuyan Zhang, Guomin Rao, Xuehua Ge, Kun Zhang, Jianfeng Li, Xiaohui Liu, Qianchao Wang, Ting Lin, Ling Cheng, Mengjiao Zhu, Xiaotang Wu, Dongchun Wang

**Affiliations:** 1 The Fourth Department of Neurosurgery, Tangshan Gongren Hospital, Tangshan, Hebei, China; 2 The Fourth Department of Neurology, Tangshan Gongren Hospital, Tangshan, Hebei, China; 3 Department of Thoracic Surgery, Tangshan People’s Hospital, Tangshan, Hebei, China; 4 Department of Breast Surgery, Tangshan People’s Hospital, Tangshan, Hebei, China; 5 Research Assistant, Shanghai Engineering Research Center of Pharmaceutical Translation, Shanghai, China

**Keywords:** glioma, HAS2-AS1, invasion and migration, USF1

## Abstract

**Objective:** The role of lncRNAs in tumor has been widely concerned. The present study took HAS2-AS1 (the antisense RNA 1 of HAS2) as a starting point to explore its expression in glioma and its role in the process of migration and invasion, providing a strong theoretical basis for mining potential therapeutic targets of glioma.

**Methods:** Clinical data of glioma were obtained from The Cancer Genome Atlas (TCGA) database and differentially expressed lncRNAs were analyzed by edgeR. The hTFtarget database was used to predict the upstream transcription factors of HAS2-AS1 and the JASPAR website was used to predict the binding sites of human upstream transcription factor 1 (USF1) and HAS2-AS1. qRT-PCR was used to detect the expressions of HAS2-AS1 and USF1 in glioma tissues and cell lines. The effects of silencing HAS2-AS1 on the migration and invasion of cancer cells were verified by wound healing and Transwell invasion assays. The chromatin immunoprecipitation (ChIP) and dual luciferase reporter assays were applied to demonstrate the binding of USF1 and HAS2-AS1 promoter region. Western blot was used to detect the expressions of epithelial–mesenchymal transition (EMT)-related proteins.

**Results:** HAS2-AS1 was highly expressed in glioma tissues and cells, and was significantly associated with poor prognosis. Silencing HAS2-AS1 expression inhibited glioma cell migration, invasion and EMT. USF1 was highly expressed in glioma and positively correlated with HAS2-AS1. The transcription of HAS2-AS1 was activated by USF1 via binding to HAS2-AS1 promoter region, consequently potentiating the invasion and migration abilities of glioma cells.

**Conclusion:** These results suggested that the transcription factor USF1 induced up-regulation of lncRNA HAS2-AS1 and promoted glioma cell invasion and migration.

## Introduction

Malignant glioma accounts for approximately 60% of human malignant primary brain tumors, and is one of the most harmful tumors in intracranial tumors, seriously affecting the health of patients [1]. According to the World Health Organization (WHO) classification, glioma can be divided into four grades: I, II for low-grade glioma (LGG), III, and IV for high-grade glioma (HGG), among which the malignant degree of glioblastoma multiforme (GBM) is the highest. At present, surgical resection is the first choice for effective treatment of glioma, along with radiotherapy, chemotherapy, immunotherapy, photodynamic therapy, electric field therapy and other auxiliary therapies. However, despite recent advances in treatment, the prognosis of glioma is still unsatisfied, especially for GBM, whose average survival is less than 1.5 years with a 5-year survival rate of 9.8% [2]. Therefore, it is very important to explore the molecular mechanism underlying the development of glioma and to find out the potential diagnostic and therapeutic targets to improve the survival rate of patients.

In recent years, more and more studies have focused on lncRNAs as tumor diagnostic markers and potential therapeutic targets. Numerous researches have shown that lncRNAs play an important regulatory role in the development of various cancers, including glioma [3–5]. For example, linc00319 expresses at a high level in glioma and is significantly associated with poor prognosis of glioma patients, while knockdown of linc00319 impairs cell proliferation, arrests cell cycle and induces cell apoptosis of glioma cells [6]. Linc00645 promotes TGF-β-induced epithelial–mesenchymal transition (EMT) by regulating the miR-205-3p-ZEB1 axis in glioma [7]. However, the regulatory mechanisms of multiple lncRNAs in glioma have not been studied yet.

HAS2-AS1 is the antisense RNA 1 of HAS2 on chromosome 8. In human aortic smooth muscle cells, HAS2-AS1 induces transcription of *HAS2* gene by recruiting transcription factors to the promoter [8]. Related studies have found that HAS2-AS1 is abnormally expressed in a variety of cancers, such as epithelial ovarian cancer [9], breast cancer [10], non-small cell lung cancer [11] and oral squamous cell cancer [12], and induces the development of cancers. Silencing of HAS2-AS1 mediates PI3K/Akt signaling pathway to inhibit cell proliferation, migration, and invasion of glioma [13]. However, the mechanism of the high expression of HAS2-AS1 in glioma is still unknown. Therefore, in-depth study on the mechanism of HAS2-AS1 will help us further explore the pathogenesis of glioma.

For the past few years, several studies have indicated that transcription factors can influence the occurrence of diseases by regulating gene expression [14,15]. Human upstream transcription factor 1 (USF1) gene is located in region q22.3 of chromosome 1 and contains a helix–loop–helix leucine zipper structure. It binds to E-BOX in various gene promoter regions and is involved in regulating the genes associated with lipid and carbohydrate homeostasis in the body [16,17]. Here, this study mined the transcription factor USF1 of HAS2-AS1 through bioinformatics, and explored the influence of HAS2-AS1 on the invasion and migration of glioma cells, which provided a new theoretical basis for the targeted treatment of glioma.

## Materials and methods

### Bioinformatics analysis

The hTFtarget database (http://bioinfo.life.hust.edu.cn/hTFtarget) was used to predict transcription factors of HAS2-AS1. The binding sites of USF1 and HAS2-AS1 were predicted by JASPAR website (http://jaspar.genereg.net/sites/MA0093.2/).

### Clinical sample collection

Glioma tissue samples of neurosurgical patients in Tangshan Gongren Hospital were collected (*n*=20). Among these patients, there were 14 LGG tissue samples (LGGTs; *n*=14) and 6 HGG tissue samples (HGGTs, *n*=6). Meanwhile, normal brain tissue samples were collected from 20 patients with acute craniocerebral injury. All tissue samples were frozen in liquid nitrogen immediately after surgical resection and stored at −80°C until use. All patients had signed informed consent, and the present study was approved by the ethics committee of Tangshan Gongren Hospital. All the patients were clinically classified according to the classification of tumors issued by WHO in 2007, and they had not been treated with chemotherapy, radiotherapy, biotherapy or other targeted therapies before the visit.

### Cell culture and transfection

Human astrocytes HA cell line (BNCC341796) was cultured in RPMI-1640 medium (Gibco, Grand Island, NY, U.S.A.) with 10% fetal bovine serum (FBS; Gibco, Carlsbad, CA, U.S.A.). Human glioma cell lines U87 (BNCC352184) and U251 (BNCC337874) were cultured in Dulbecco’s modified Eagle’s medium (DMEM)/hyperglycemia with 10% FBS. All cell lines were purchased from BeNa Culture Collection (China). The cells were all cultured at 37°C and stored in a constant incubator with 5% CO_2_.

Si-HAS2-AS1, si-USF1, oe-HAS2-AS1 and their negative control lentivirus packaging vectors were purchased from Invitrogen (Carlsbad, CA, U.S.A.). After at least 24 h of culture, the plasmids were transfected into glioma cells by Lipofectamine 2000 (Thermo Fisher Scientific, Inc.) according to the manufacturer’s instructions.

### RNA extraction and qRT-PCR

Total RNA from tissues and cells was extracted using TRIzol Reagent (Invitrogen), while RNA quality and concentration were measured using a Nanodrop 1000 spectrophotometer (Thermo Fisher Scientific, Inc.) according to the manufacturer’s specifications. cDNA was prepared by reverse transcription kit (Invitrogen). Applied Biosystems 7300 Real-Time PCR System (Applied Biosystems, U.S.A.) was used for qRT-PCR. The primers used were purchased from Sangon Biotech Co., Ltd. (Shanghai, China), as shown in Supplementary Table S1. Glyceraldehyde-3-phosphate dehydrogenase (GAPDH) was used as an internal regulator, and the differences in gene expression of the control group and experimental group were compared by 2^−ΔΔ*C*_t_^ method.

### Transwell

Approximately 2 × 10^4^ cells were added to Transwell (BD Biosciences) upper chamber, which was coated with Matrigel matrix (Corning, Corning, NY). The lower chamber was filled with DMEM containing 10% FBS. After incubation at 37°C for 24 h, cells that did not pass through the membrane were removed with a cotton swab. While the cells in the lower chamber were fixed with 4% paraformaldehyde, and stained with 0.4% Crystal Violet. Then, four fields were randomly selected and observed under a microscope to count the number of cells that successfully invaded the matrix glue. The experiment was repeated three times.

### Wound healing assay

The wound healing assay was performed to measure cell migration. A pipette was used to create scratched cell-free areas when cells grew approximately 70–80% in confluence in the culture wells. The wells were briefly washed twice with medium to remove the separated cells. After 48 h of growth with fresh medium, the cells were photographed under a microscope and the migration rates of U87 and U251 cells were calculated, respectively.

### Western blot

Total proteins were extracted and protein concentration was determined using a BCA kit (Thermo, U.S.A.). A total of 30 μg of total proteins were loaded on to SPS/PAGE and transferred on to PVDF membrane (Amersham, U.S.A.) after electrophoresis. The membrane was blocked with 5% skim milk powder at room temperature for 1 h and after that the blocking buffer was discarded. Then the membrane was cultured with primary antibodies at 4°C overnight and washed with PBST 10 min for three times. Next the horseradish peroxidase-labeled secondary antibody was added and the membrane was incubated at room temperature for 1 h and washed with PBST following the above steps. After scanning and developing with an optical luminescence instrument (GE, U.S.A.), Image-Pro Plus 6.0 (Media Cybernetics, U.S.A.) software was used to conduct grayscale scanning of the protein bands to analyze the relative protein expression. The experiment was repeated three times.

The primary antibodies used in the experiment were N-cadherin rabbit polyclonal antibody (ab76057, 1:1000, Abcam, Cambridge, U.K.), E-cadherin rabbit polyclonal antibody (ab15148, 1:500, Abcam, Cambridge, U.K.), Vimentin rabbit polyclonal antibody (ab137321, 1:2000, Abcam, Cambridge, U.K.) and GADPH rabbit polyclonal antibody (ab9485, 1:2500, Abcam, Cambridge, U.K.). The secondary antibody was goat anti-rabbit IgG H&L (HRP) (ab6721, 1:300, Abcam, Cambridge, U.K.).

### Chromatin immunoprecipitation assay

Chromatin immunoprecipitation (ChIP) assay was performed using the Simple ChIP Enzymatic Chromatin IP kit (Cell Signaling Technology, U.S.A.) according to the manufacturer’s instructions. Cells (U87 and U251) were harvested for cross-linking reaction with 1% formaldehyde in the medium for 10 min and then quenched with glycine at room temperature for 5 min. These cells were then lysed in lysis buffer and sequentially collected. With 2% lysates as input, the other lysates were rotational incubated with normal rabbit IgG or anti-USF1 antibodies. DNA cross-linking was reversed by NaCl and protease K to purify DNA. The DNA level of HAS2-AS1 was measured by qRT-PCR.

### Construction of luciferase report vector

The binding sites of USF1 on the HAS2-AS1 promoter region were cloned into restriction enzymes Kpnl and HindIII digestion sites 200 on firefly luciferase reporter vector pGL3 (Promega, Madison, WI, U.S.A.). According to the manufacturer’s instructions, the above vectors and the USF1 expression vector (USF1 construct) or the negative control (empty vector) were co-transfected into U87 and U251 cells instantaneously by Lipofectamine 2000 (Invitrogen). The relative luciferase activity was evaluated using a dual luciferase reporting system (Promega, Madison, WI, U.S.A.) 48 h after transfection.

### Statistical analysis

SPSS 21.0 statistical software (SPSS; Inc., Chicago, IL, U.S.A.) was used to analyze the data. The measurement data were expressed as mean ± standard deviation. *t* test was used for analyzing comparisons between two groups, while comparisons among multiple groups were analyzed by using one-way analysis of variance (ANOVA) with Tukey’s post hoc test. The correlation analysis was conducted using Pearson’s correlation analysis. *P*<0.05 indicated statistically significant difference, while *P*<0.01 indicated extremely significant difference.

## Results

### Silencing HAS2-AS1 inhibits glioma cell invasion and migration

**Figure 1:**
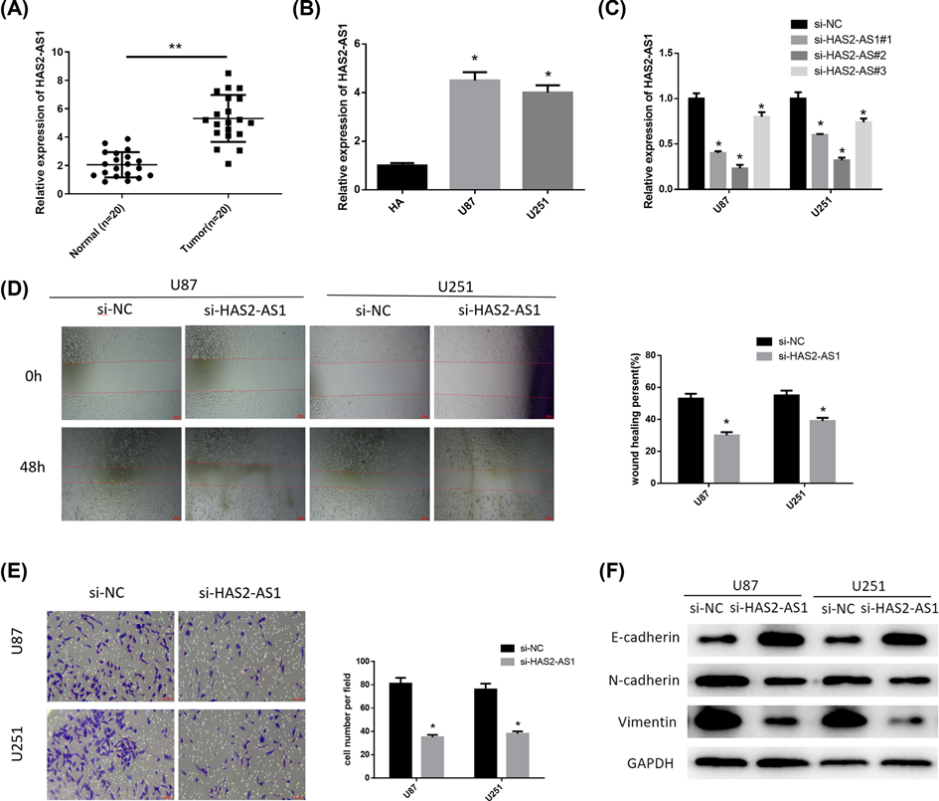
Silencing HAS2-AS1 expression inhibits glioma cell invasion and migration (**A**) The expression of HAS2-AS1 in glioma tissues and normal tissues (*n*=20). (**B**) The expression of HAS2-AS1 in normal human astrocytes HA and glioma cells U87 and U251 were detected by qRT-PCR. (**C**) qRT-PCR was used to detect the interference efficiency of three siRNAs (si-HAS2-AS1#1, si-HAS2-AS1#2, si-HAS2-AS1#3). (**D**) The migration ability of glioma cells after silencing HAS2-AS1 was detected by wound healing assay. (**E**) The invasion ability of glioma cells after silencing HAS2-AS1 was detected by Transwell assay. (**F**) The expressions of EMT-related proteins E-cadherin, N-cadherin and Vimentin were detected by Western blot. *P<0.05, **P<0.01.

Multiple studies have reported that HAS2-AS1 is involved in the regulatory mechanism of ceRNA network and modulates the proliferation and migration of tumor cells [13,18]. Here, HAS2-AS1 was chosen for follow-up experiments. Our result shows that HAS2-AS1 was significantly highly expressed in either tumor tissues (Figure 1A) and glioma cell lines U87 and U251 (Figure 1B). In order to verify whether HAS2-AS1 was a functional target of glioma, we interfered the expression of HAS2-AS1 in U87 and U251 cell lines, and detected the invasion and migration abilities of glioma cells. First, we tested the silencing efficiency of three si-RNAs, and si-HAS2-AS1#2 with the highest silencing efficiency was selected for the following cell experiments (Figure 1C).

The results of wound healing (Figure 1D) and Transwell (Figure 1E) assays exhibited that after 48 h, the migration and invasion abilities of glioma cells in the si-HAS2-AS1 group were significantly reduced compared with those in the si-NC group. Then, we further detected the expression of EMT-related proteins in the cells of each group by Western blot. Tumor migration is always accompanied by EMT, which exhibits decreased E-cadherin expression along with increased N-cadherin and Vimentin expressions. It could be seen from the results that silencing HAS2-AS1 inhibited EMT of glioma cells (Figure 1F). These results indicated that silencing HAS2-AS1 could inhibit the invasion and migration of glioma cells.

### USF1 activates the expression of HAS2-AS1 by binding to the HAS2-AS1 promoter region

**Figure 2:**
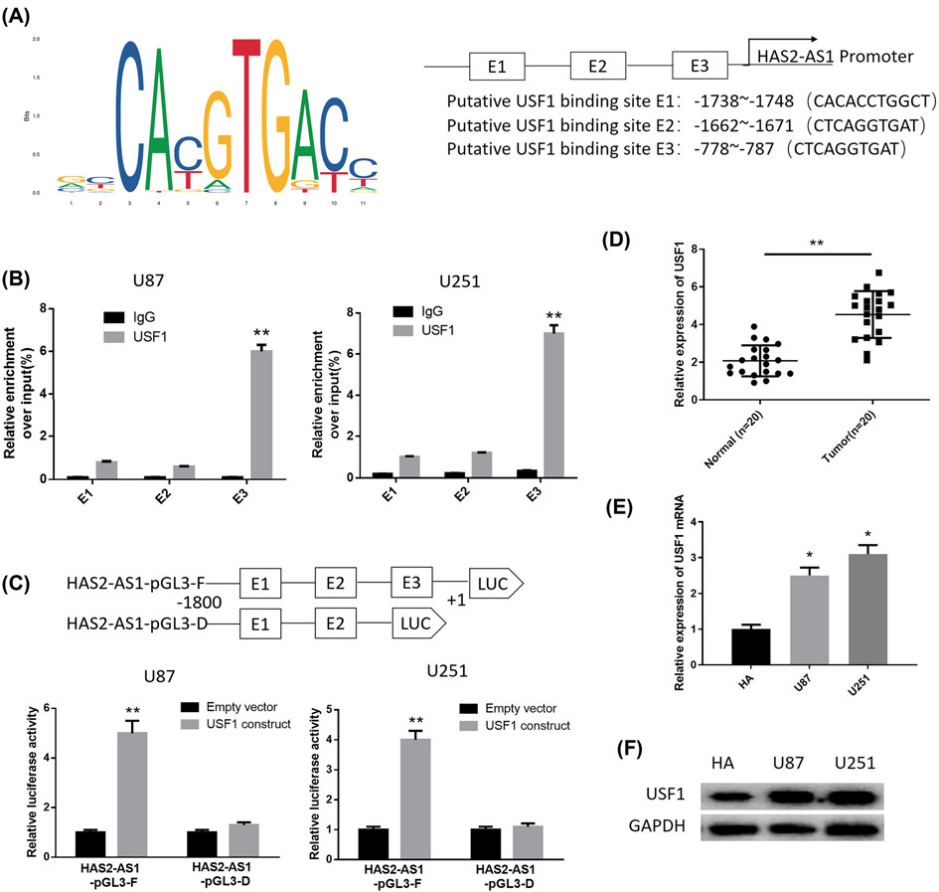
USF1 is highly expressed in glioma and activates the transcription of HAS2-AS1 by binding to the HAS2-AS1 promoter region (**A**) The binding sites of USF1 on HAS2-AS1 promoter region and the first three binding sequences were obtained using the JASPAR website. (**B**) ChIP assay verified the reliability of the three binding sequences of USF1 on HAS2-AS1 promoter region. (**C**) Luciferase reporter assay verified that E3 segment was bound to USF1. (**D**) qRT-PCR was used to detect the expression of USF1 in glioma tissues and normal tissues (*n*=20). (**E**) The expression of USF1 in normal human astrocytes HA and glioma cells U87 and U251 were detected by qRT-PCR. (**F**) Western blot was used to detect the protein expression of USF1 in each group. *P<0.05, **P<0.01.

Based on the above results, we found that the up-regulation of HAS2-AS1 was related to the development of glioma, thus, it is necessary to explore the underlying molecular mechanism. Several studies have demonstrated that lncRNAs can be activated by their upstream transcription factors [15,19,20]. The transcription factors that regulate the expression of HAS2-AS1 were predicted through the hTFtarget database, and USF1 was selected as the object. It was found that the expression of USF1 in the tumor group was significantly up-regulated and positively correlated with the expression of HAS2-AS1 (Supplementary Figure S1). In addition, USF1 has been found to regulate the development of various cancers [21–23], but whether it can regulate the transcription of HAS2-AS1 remains unclear. Based on the correlation analysis and research status, we chose USF1 as the research object. To verify that HAS2-AS1 was activated by USF1 in glioma cells, we obtained the binding sites and the first three binding sequences of USF1 on HAS2-AS1 promoter region using the JASPAR website (Figure 2A). ChIP assay was used to verify the reliability of the predicted sites, as displayed in Figure 2B. E3, rather than E1 or E2, was the binding site of USF1 on the HAS2-AS1 promoter region. In addition, results of luciferase reporter assay showed that the −1800 to +1 region of the HAS2-AS1 promoter drove luciferase activity, while luciferase activity was decreased with the absence of E3 fragment (Figure 2C). All of these findings suggested that the transcription of HAS2-AS1 was most likely to be activated by USF1 via binding to the HAS2-AS1 promoter region.

Then, we further detected the expression of USF1 in glioma tissues and cells by qRT-PCR (Figure 2D,E). The results illustrated that USF1 was significantly highly expressed in glioma tissues and cells. The protein expression of USF1 was detected by Western blot and it was found to be highly expressed in glioma cell lines U87 and U251 (Figure 2F). These results indicated that the abnormal high expression of USF1 in glioma cells may be one of the causes for the high expression of HAS2-AS1.

### Silencing USF1 inhibits glioma cell invasion and migration by inhibiting the expression of HAS2-AS1

**Figure 3:**
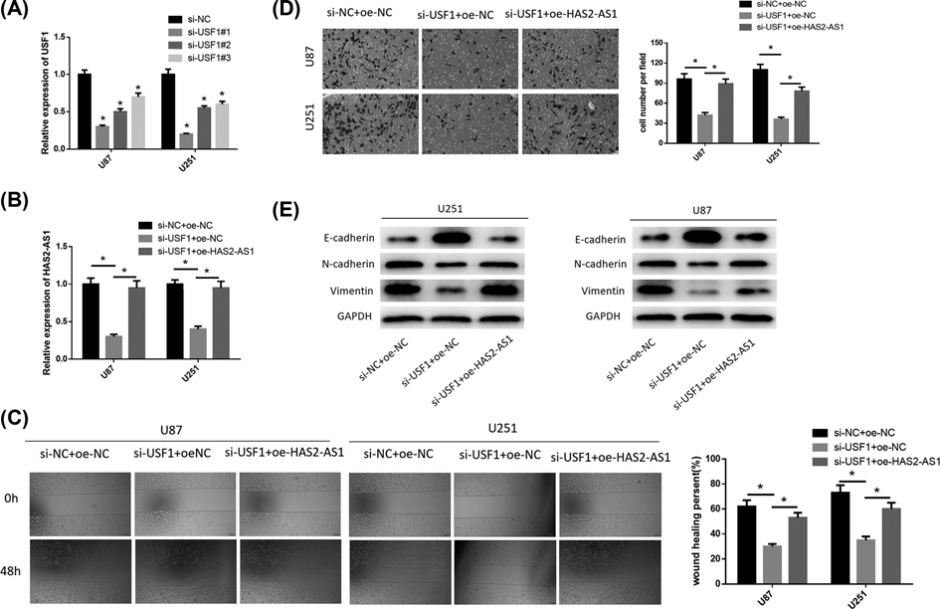
Silencing USF1 inhibits glioma cell invasion and migration by inhibiting the expression of HAS2-AS1 (**A**) qRT-PCR was performed to detect the interference efficiency of three siRNAs (si-USF1#1, si-USF1#2, si-USF1#3); (**B**) The expression of HAS2-AS1 in si-NC+oe-NC, si-USF1+oe-NC, si-USF1+oe-HAS2-AS1 groups were detected by qRT-PCR. (**C**) Cell migration in each group was detected by wound healing assay. (**D**) Cell invasion ability in each groups was detected by Transwell assay. (**E**) The expression of EMT-related proteins E-cadherin, N-cadherin and Vimentin in each group were detected by Western blot. *P<0.05.

To verify the effects of USF1 on glioma invasion and migration, we silenced USF1 and overexpressed HAS2-AS1 expression in glioma cell lines U87 and U251. First, we tested the interference efficiency of three siRNAs against USF1, and found that si-USF1#1 had the highest interference efficiency. Therefore, si-USF1#1 was transfected into cells for the following experiments (Figure 3A). Then, we divided the two glioma cell lines into three groups: si-NC+oe-NC, si-USF1+oe-NC, and si-USF1+oe-HAS2-AS1. The expression of HAS2-AS1 was detected by qRT-PCR. It was observed that silencing USF1 inhibited the expression of HAS2-AS1, but the results were reversed after the simultaneous transfection of HAS2-AS1 overexpression vector (Figure 3B). Subsequently, we measured the migration and invasion abilities of cells through wound healing (Figure 3C) and Transwell assays (Figure 3D). The results showed that silencing USF1 dramatically inhibited migration and invasion abilities of glioma. However, overexpression of HAS2-AS1 restored cell invasion and migration abilities to some extent. Finally, we detected the expressions of EMT-related proteins (Figure 3E). Compared with the negative control group, the expression of E-cadherin was increased, while the expressions of N-cadherin and Vimentin were down-regulated in the si-USF1+oe-NC group, indicating that EMT was inhibited. However, after we overexpressed HAS2-AS1, EMT was recovered. These results indicated that silencing USF1 could inhibit the expression of HAS2-AS1, thus inhibiting the invasion and migration of glioma cells.

## Discussion

In glioma, more and more functions of lncRNAs have been explored, and the molecular mechanism of abnormal expression of these lncRNAs in cancer is one of the hot topics of current researches. In the present study, it was proved that HAS2-AS1 was significantly highly expressed in glioma. Related studies have exhibited that HAS2-AS1 can regulate the malignant progression of various cancers. For example, CAMP-responsive element binding protein 1 (CREB1)-induced HAS2-AS1 promotes epithelial ovarian cancer cell proliferation and invasion via the miR-466/Runx2 axis [9]. HAS2-AS1 mediates hypoxia-induced invasiveness of oral squamous cell carcinoma [12]. In GBM, the reduction in HAS2-AS1 inhibits GBM cells migration and invasion *in vitro* and *in vivo* [24]. Therefore, we speculated that HAS2-AS1 might play a role as an oncogene in glioma.

To test this hypothesis, we collected clinical tumor tissue and normal tissue samples from 20 glioma patients, and found that HAS2-AS1 was significantly up-regulated in tumor tissues, while similar results were obtained in glioma cell lines. We further investigated the effects of HAS2-AS1 on glioma invasion and migration through cell experiments. The results discovered that silencing HAS2-AS1 significantly inhibited the migration and invasion of cancer cells. Meanwhile, the expressions of EMT-related proteins in each group further confirmed the above result, and silencing HAS2-AS1 inhibited the occurrence of EMT. These studies fully demonstrated that silencing HAS2-AS1 could inhibit the development of glioma, and HAS2-AS1 might be a potential target for glioma treatment.

The expression of HAS2-AS1 can be regulated by many transcription factors in a variety of cancers including glioma, such as CREB1 [25], hypoxia-inducible factor 1α (HIF-1α) [26], signal transducer and activator of transcription 1 (STAT1) [23] etc. However, gene expression is regulated by many factors, so it is of great significance to further explore new transcription factors to construct a complete regulatory network. To this end, we used bioinformatics to explore the upstream transcription factors of HAS2-AS1, and found that the expression of USF1 in the tumor group was significantly up-regulated and positively correlated with the expression of HAS2-AS1. Until now, no studies have shown that USF1 can regulate the transcription of HAS2-AS1. Recent studies have found that USF1 is involved in regulating the development of various types of tumors. In oral mucosal cancer, USF1 regulates human telomerase gene expression, which leads to telomerase reactivation and oral carcinogenesis [16]. The up-regulation of LINC01048 induced by USF1 promotes cell proliferation and apoptosis in cutaneous squamous cell carcinoma by binding to TAF15 to activate YAP1 transcription [20]. In glioma, USF1 can increase blood–brain barrier permeability and down-regulate the expression of tight junction protein in glioma microvascular endothelial cells [27]. Moreover, Di Wang et al*.* found that knockout of USF1 inhibits the vasculogenic mimicry of glioma cells via stimulating small nucleolar RNA host gene 16 (SNHG16)/miR-212-3p and Linc00667/miR-429 axis [28]. In this study, we verified that USF1 could bind to the HAS2-AS1 promoter region (CTCAGGCTAT) to activate the transcription of HAS2-AS1 through ChIP and luciferase assays. At the same time, we proved that USF1 was significantly highly expressed in glioma tumor tissues and cell lines. Inhibiting the expression of USF1 could reduce the migration and invasion abilities of glioma cells, while overexpressing HAS2-AS1 could recover the inhibitory effects of USF1 on glioma cells to a certain extent.

In summary, our results demonstrated that both HAS2-AS1 and USF1 were highly expressed in glioma tissues and cells, and USF1 could bind to the HAS2-AS1 promoter region to activate the transcription of HAS2-AS1, thus promoting the migration and invasion of glioma cells. The present study revealed the mechanism of high HAS2-AS1 expression in glioma, and as a tumor promoter, HAS2-AS1 might be a potential diagnostic marker and a new therapeutic target for glioma.

## Highlights

1. The expressions of USF1 and HAS2-AS1 are positively correlated, and both are significantly up-regulated in glioma.
2. The transcription factor USF1 activates the transcription of HAS2-AS1 by binding to the promoter region of HAS2-AS1.
3. USF1 promotes glioma invasion and migration by activating HAS2-AS1.

## Supplementary Material

Supplementary Figure S1 and Table S1

## Abbreviations

CAMP: cathelicidin antimicrobial peptide
cDNA: complementary DNA
ceRNA: competing endogenous RNAs
ChIP: chromatin immunoprecipitation assay
CREB1: CAMP-responsive element binding protein 1
DMEM: Dulbecco’s modified Eagle’s medium
EMT: epithelial–mesenchymal transition
FBS: fetal bovine serum
GAPDH: glyceraldehyde-3-phosphate dehydrogenase
GBM: glioblastoma multiforme
HAS2-AS1: the antisense RNA 1 of HAS2
HGG: high-grade glioma
HGGTs: HGG tissue samples
LGG: low-grade glioma
LGGTs: LGG tissue samples
qRT-PCR: Quantitative real time polymerase chain reaction
SNHG16: small nucleolar RNA host gene 16
STAT1: signal transducer and activator of transcription 1
TAF15: TATA-box binding protein associated factor 15
TCGA: The Cancer Genome Atlas
USF1: human upstream transcription factor 1
WHO: World Health Organization
YAP1: Yes1 associated transcriptional regulator
