# Supplementary material for: The transcription factor USF1 promotes glioma cell invasion and migration by activating lncRNA HAS2-AS1
Source: Biosci Rep. 2020 Aug 21;40(8):BSR20200487. doi: 10.1042/BSR20200487 (PMC7442972; doi:10.1042/BSR20200487)
Supplement: Supplementary Figure S1 and Table S1 [file BSR-2020-0487_supp.pdf]

A

gene1	gene2	Cor	pvalue
HAS2-AS1	MAX	0.223	2.64E-09
HAS2-AS1	SPI1	0.399	5.33E-28
HAS2-AS1	RAD21	0.334	1.33E-19
HAS2-AS1	MED1	0.201	9.16E-08
HAS2-AS1	TAF1	0.128	0.00069
HAS2-AS1	MXI1	0.119	0.001572
HAS2-AS1	SIN3A	0.184	9.41E-07
HAS2-AS1	EP300	0.07	0.063817
HAS2-AS1	TCF12	0.176	3.01E-06
HAS2-AS1	POLR2A	0.017	0.648564
HAS2-AS1	CTCF	0.165	1.22E-05
HAS2-AS1	SMC3	0.132	0.000467
HAS2-AS1	USF1	0.259	3.47E-12
HAS2-AS1	GATA3	0.206	3.96E-08

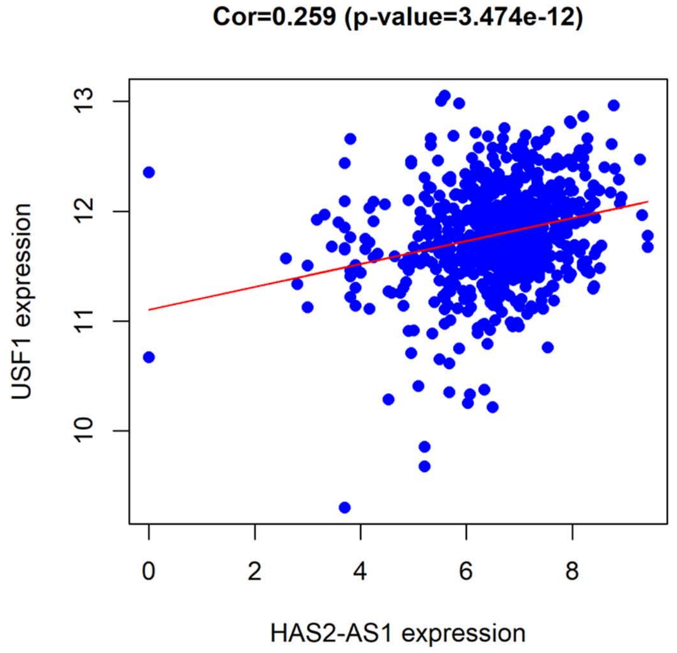

B

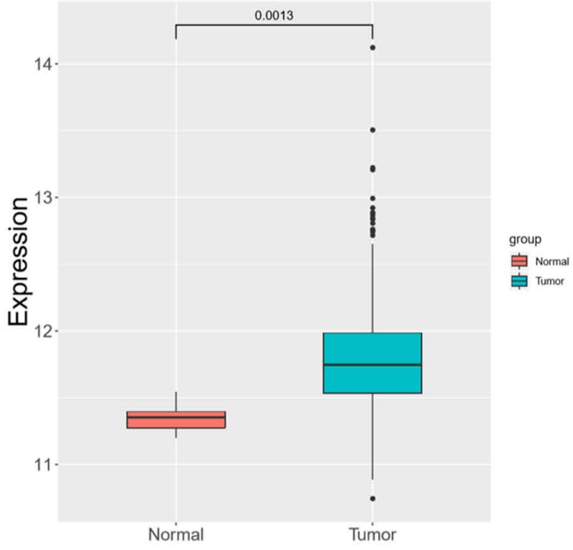

Supplement Figure 1 USF1 is highly expressed in glioma and is positively correlated with the expression of HAS2-AS1

(A) Correlation analysis of the transcription factor USF1 and HAS2-AS1; (B) Relative expression of USF1 glioma tumor and normal tissues.

Supplement Figure 2 The full uncropped and unedited versions of Western blots

Supplement Table 1 Primer sequences for qRT-PCR

Genes	Primer sequences
HAS2-AS1	F:5'-AGGGGTGGACTTCTTTGGAAC-3'<br>R:5'-CCAAACAGCTCCTTGTGCG-3'
USF1	F:5'-CACTAAACTCTGGGGCTTGTCC-3'<br>R:5'-CACCAGCCACTGCTAAACATCC-3'
GAPDH	F:5'-GGAGCGAGATCCCTCCAAAAT-3'<br>R:5'-GGCTGTTGTCATACTTCTCATGG-3'
